# Guidelines for Burr Hole Surgery in Combination With Tumor Treating Fields for Glioblastoma: A Computational Study on Dose Optimization and Array Layout Planning

**DOI:** 10.3389/fnhum.2022.909652

**Published:** 2022-06-16

**Authors:** Fang Cao, Nikola Mikic, Eric T. Wong, Axel Thielscher, Anders Rosendal Korshoej

**Affiliations:** ^1^Department of Health Technology, Center for Magnetic Resonance, Technical University of Denmark, Kgs. Lyngby, Denmark; ^2^Danish Research Centre for Magnetic Resonance, Centre for Functional and Diagnostic Imaging and Research, Copenhagen University Hospital Amager and Hvidovre, Hvidovre, Denmark; ^3^Department of Neurosurgery, Aarhus University Hospital, Aarhus, Denmark; ^4^Department of Clinical Medicine, Aarhus University, Aarhus, Denmark; ^5^Division of Hematology/Oncology, Department of Medicine, Rhode Island Hospital, Providence, RI, United States

**Keywords:** computational modeling, tumor treating fields (TTFields), skull remodeling surgery, dose, burr hole, glioblastoma, array layout, SimNIBS

## Abstract

Tumor treating fields (TTFields) is an anti-cancer technology increasingly used for the treatment of glioblastoma. Recently, cranial burr holes have been used experimentally to enhance the intensity (dose) of TTFields in the underlying tumor region. In the present study, we used computational finite element methods to systematically characterize the impact of the burr hole position and the TTFields transducer array layout on the TTFields distribution calculated in a realistic human head model. We investigated a multitude of burr hole positions and layouts to illustrate the basic principles of optimal treatment planning. The goal of the paper was to provide simple rules of thumb for physicians to use when planning the TTFields in combination with skull remodeling surgery. Our study suggests a number of key findings, namely that (1) burr holes should be placed directly above the region of interest, (2) field enhancement occurs mainly underneath the holes, (3) the ipsilateral array should directly overlap the holes and the contralateral array should be placed directly opposite, (4) arrays in a pair should be placed at far distance and not close to each other to avoid current shunting, and finally (5) rotation arrays around their central normal axis can be done without diminishing the enhancing effect of the burr holes. Minor deviations and adjustments (<3 cm) of arrays reduces the enhancement to some extent although the procedure is still effective in these settings. In conclusion, our study provides simple guiding principles for implementation of dose-enhanced TTFields in combination with burr-holes. Future studies are required to validate our findings in additional models at the patient specific level.

## Introduction

**Figure 1:**
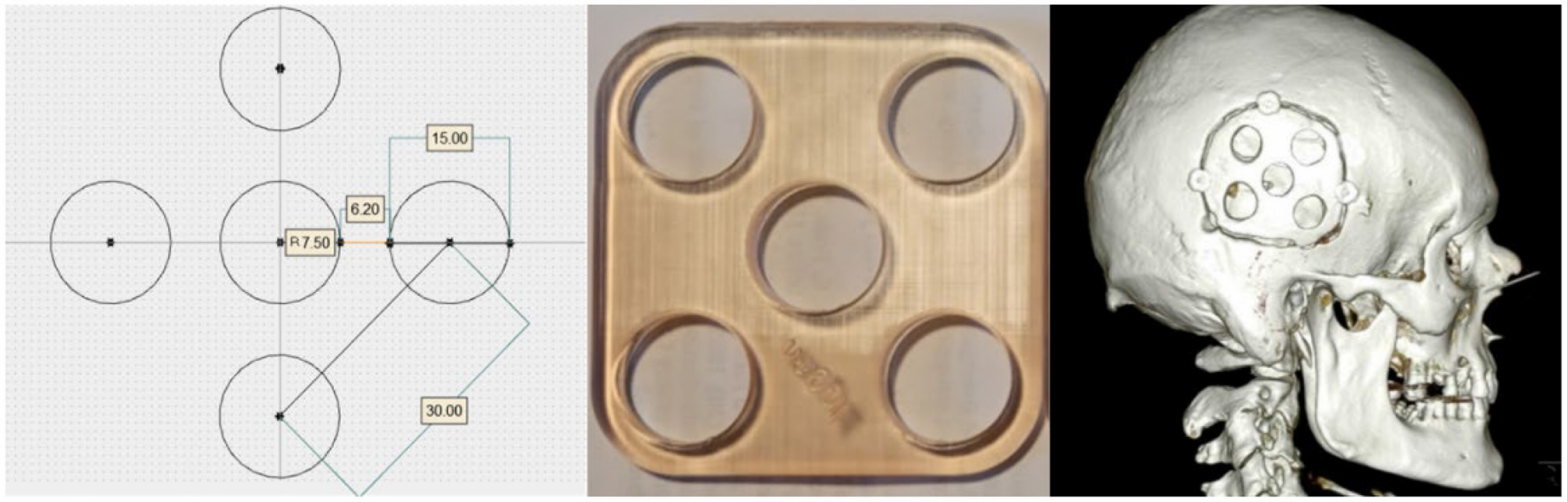
Illustrates the quinconce configuration and the 3D printed template used preoperatively to aid in creating the configuration. The 3D CT scan of the cranium shows the burr hole quinonce configuration in a trial participant.

Tumor treating fields (TTFields) therapy is an anti-cancer technology that induces low-intensity fields locoregionally to inhibit tumor growth. The technology is increasingly being used to treat a multitude of solid cancers (Novocure, 2021). Currently, TTFields is a Category 1A recommendation for the treatment of newly diagnosed glioblastoma brain cancer in the US (Nabors et al., 2020). One important factor that has been identified to influence the efficacy of the TTFields therapy is the electric field strength. High electric field intensities reduce the rate of tumor cell division *in vitro* (Kirson et al., 2004) and increase progression-free survival (PFS) and overall survival (OS) in GBM patients (Wenger et al., 2018; Ballo et al., 2019). Recently, it was proposed to introduce holes at strategic positions in the skull (skull-remodeling surgery, SR-surgery) to facilitate current flow into the tumor, and thereby enhance the anti-neoplastic dose of TTFields focally in the region of interest (Korshoej et al., 2016). The principle has been described and analyzed in preclinical modeling studies (Korshoej et al., 2018; Mikic and Korshoej, 2021) and the concept translated into a phase 1 clinical trial demonstrating safety in 15 patients with recurrent GBM (Korshoej et al., 2020). Furthermore, the trial indicated prolonged overall survival (15.5 months) relative to 6–11 months in comparable first recurrence GBM trial population (Taal et al., 2014) and showed that an average of 32% (range 25–59%) field enhancement could be obtained with an mean skull defect area of 10.5 cm^2^ (range 7–48 cm^2^). Currently, an ongoing Scandinavian multinational phase 2 trial (NCT0422399) aims to investigate the efficacy of the intervention in a randomized comparative setting with 84 patients (Mikic et al., 2021). The patients are randomized 1:1 to receive standard medical oncological treatment and TTFields w/o skull-remodeling therapy and the primary endpoint is overall survival rate at 12 months. In this trial, burr holes are applied as five 15 mm holes arranged in a quinconce configuration (Figure 1). The rationale is that several small burrholes have been demonstrated more effective than a single craniectomy of equivalent area (Korshoej et al., 2016).

In this paper, we analyze the impact of various positions of burr-hole quinconces and associated transducer array layouts by computing the electric field intensity distributions. The aim is to provide a general and principal framework for clinicians to employ when implementing the intervention. We sought to answer basic questions raised in clinical treatment, e.g., where should the burr holes be placed, how should the arrays be placed, how can arrays be adjusted and efficient field enhancement simultaneously be maintained? Collectively, our results aim to describe and validate basic rules of thumb for SR-surgery and subsequent layout planning without the need for advanced modeling.

## Materials and Methods

### Head Model Generation

To investigate the SR-surgery configuration we used simulations of the electric fields generated by the TTFields therapy using a detailed head model constructed from structural MR images and the Finite-Element Method (FEM). The head model was initially created from a dataset of a healthy participant, which was then adapted to emulate a trial patient's pathology based on their post-operative MRI and CT of the head. More specifically, we created the computational head model using the example dataset “Ernie” in the SimNIBS software package as a proof-of-concept demonstration. The “Ernie” dataset corresponds to a young and healthy subject, including a high-resolution T1 and a T2 weighted image. Details on the “Ernie” dataset can be found in the documentation from the SimNIBS toolbox (“Ernie”).

**Step 1:** An automated tissue segmentation was performed on the T1 and T2 weighted images of the “Ernie” dataset. The initial segmentation includes the following eight tissue compartments: white matter, gray matter, CSF, scalp, skull, muscle, blood, and eyeballs.

**Figure 2:**
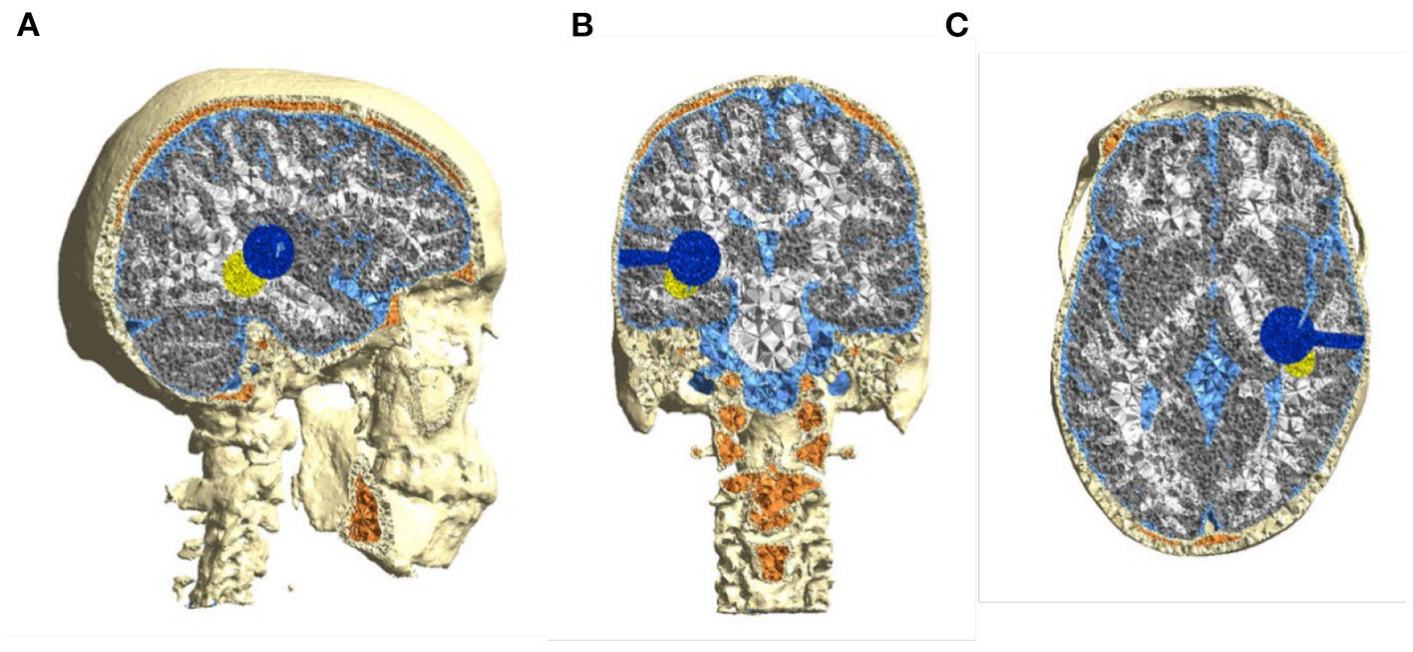
Computational head model based on healthy subject's (“Ernie”) MRI—**(A)** sagittal, **(B)** coronal, and **(C)** axial. Pathology features were inserted to mimic a real patient's postoperative MRI, i.e., a spherical 2.5 cm resection cavity and connected surgical corridor (blue) and a spherical 2.5 cm residual tumor remnant (yellow).

**Step 2:** The segmented image was visually inspected, and the configurations of the virtual tumor resection cavity, the residual tumor, and the burrholes were set manually based on the trial patient's post-operative MRI and CT of the head and were as follows:

1. A Virtual Sphere-Shaped Tumor Resection Cavity With a 2.5 cm Diameter in the “Ernie” Head Model (Figure 2).
2. A Cylinder-Shaped Funnel on the top of the Tumor Resection Cavity Mimics the Surgical Entry to the Tumor. The Funnel Track Has a 0.8 cm Diameter.
3. A Sphere-Shaped Tumor Remnant of a 2.5 cm Diameter Underneath the Tumor Resection Cavity.
4. Virtual SR-Surgery Was Applied to the Head Model. The SR Surgery Entailed Placing five Burrholes of 1.5 cm Diameter on the Skull With one Central Burr Hole Surrounded by four Evenly Distributed Burr Holes. Each Burr Hole Was Assigned CSF Conductive Value (1.654 S/m). We Investigated five Different Positions of Burrholes as Described in Section Placement of TTField Transducer Arrays.

**Step 3:** The head models were created using the segmented voxel image with virtual tumor cavity, residual tumor, and with or without burrholes. Then the voxel segmentations with/without burr holes were converted to tetrahedral head meshes using the SimNIBS toolbox. The final head mesh with each burr hole configuration consisted of a total number of ≈4,750,000 tetrahedral elements, assigned to more than 11 types, including while matter, gray matter, CSF, scalp, skull, muscle, blood, eyeballs, tumor resection cavity, residual tumor, and burrholes.

**Step 4:** The electrode arrays were placed on the skin surface for each head model as described in Section Electric Field Calculation and Evaluations. Conductivity values were assigned to the different compartments, consisting of skin (0.25 S/m), sponge bone (0.025 S/m), compact bone (0.008 S/m), CSF (1.654 S/m), gray matter (GM) (0.276 S/m), white matter (WM) (0.126 S/m), residual tumor 0.24 S/m) and necrotic tissue (1.0 S/m) (Wagner et al., 2004; Opitz et al., 2015). In addition, we merged the sponge bone and the compact bone to bone (0.010 S/m) to simplify the head model when calculating the enhancement of the electric field intensity. We created a custom version of SimNIBS and provided scripts for automated simulation of TTFields induced electric fields for variations of the electrode array positions (described in the following section).

At last, the results of the simulations were visualized using Gmsh.

### TTFields Dosimetry

The “dose” of TTFields was calculated as the field intensity, i.e., the Euclidean norm of the field vector, in concordance with many previous studies (Miranda et al., 2014; Wenger et al., 2015a,b; Korshoej et al., 2017, 2018; Lok et al., 2019, 2020). It is well-described that other factors, such as treatment exposure time, frequency, and field orientation are also important determinants of effective TTFields dose (Kirson et al., 2004; Korshoej and Thielscher, 2018a; Toms et al., 2019), however, given the rationale of skull remodeling surgery, we only considered field intensity, which was the modified parameter.

### Configurations of the Skull Remodeling Surgery

**Figure 3:**
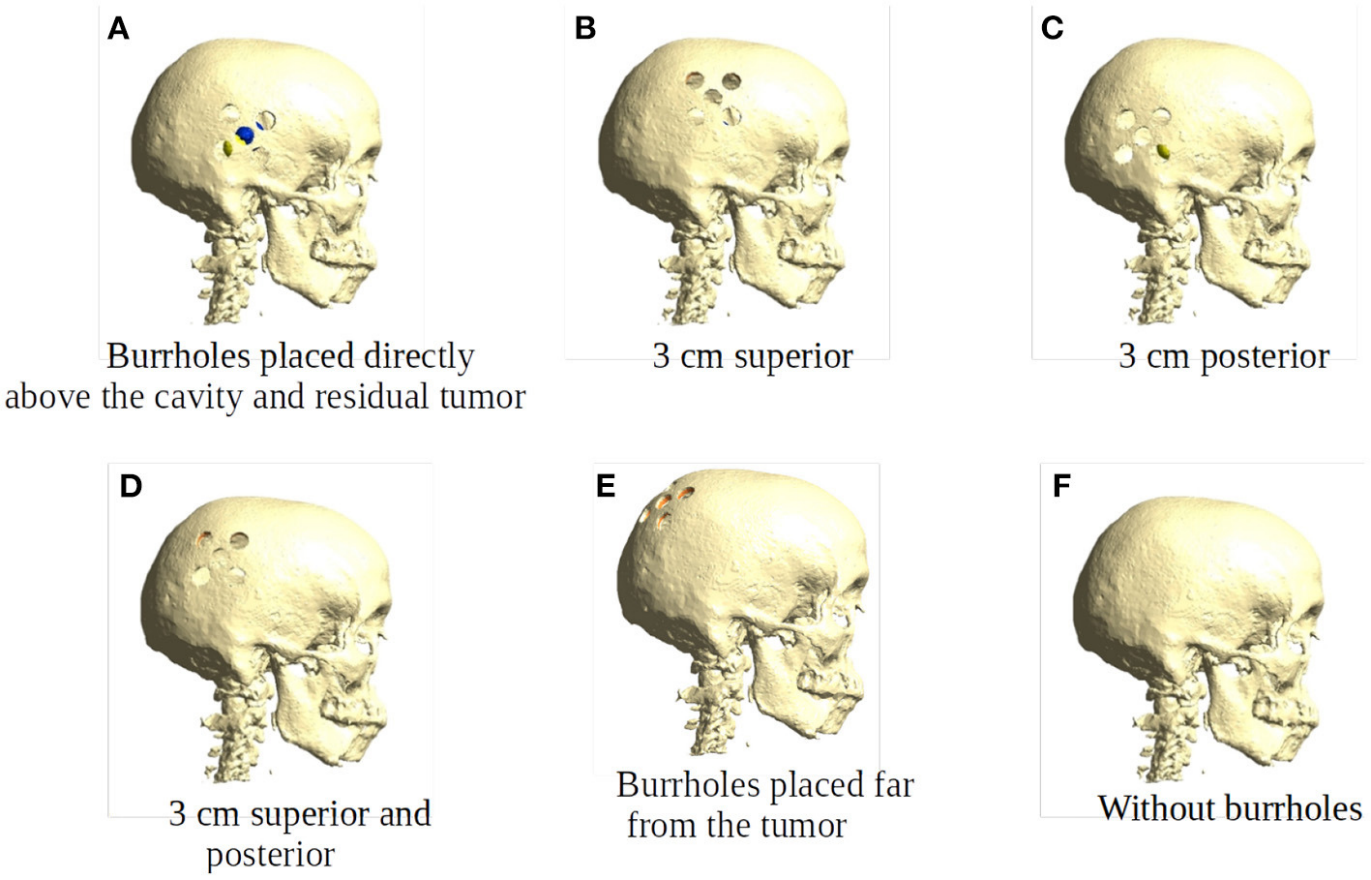
Placement of burr holes. **(A)** shows the burr holes placed directly above the resection cavity and residual tumor. **(B–E)** show the burr holes placed 3 cm superior, 3 cm posterior, 3 cm superior and posterior, and far from the resection cavity and residual tumor. **(F)** shows an intact skull that serves as the control in our experiments.

Burr-hole configurations were equivalent to those employed in the clinical trial (Mikic et al., 2021), i.e., five burr-holes of 15 mm each configured as a 45 × 45 mm quinconce, Figures 1, 3. This configuration was chosen, because previous studies have demonstrated that it is more effective to distribute the hole over a larger area to cover the underlying tumor bed, rather than creating a single large hole with an equivalent area (Korshoej et al., 2016). As a general principle, we hypothesized that the strongest field enhancement in the underlying tumor would occur if the center of the quinconce was placed directly above the resection cavity and residual tumor (Figure 3A), as this would result in the strongest flow of current into the region. This positioning was done manually in the model. To investigate the impact of suboptimal placement of burr holes, we also investigated four additional positions, namely translation of the quinconce 3 cm superior (Figure 3B), 3 cm posterior (Figure 3C), 3 cm superior and posterior (Figure 3D), and finally far from the tumor in the ipsilateral occipito-parietal region (Figure 3E). All configurations were compared to the field distributions without burr-holes (i.e., “control,” Figure 3F). The rationale for these investigations was to determine whether sufficient enhancement could still be achieved with holes placed slightly away from the tumor region and to verify that no additional enhancement would be observed with holes far away from the tumor.

### Placement of TTField Transducer Arrays

**Figure 4:**
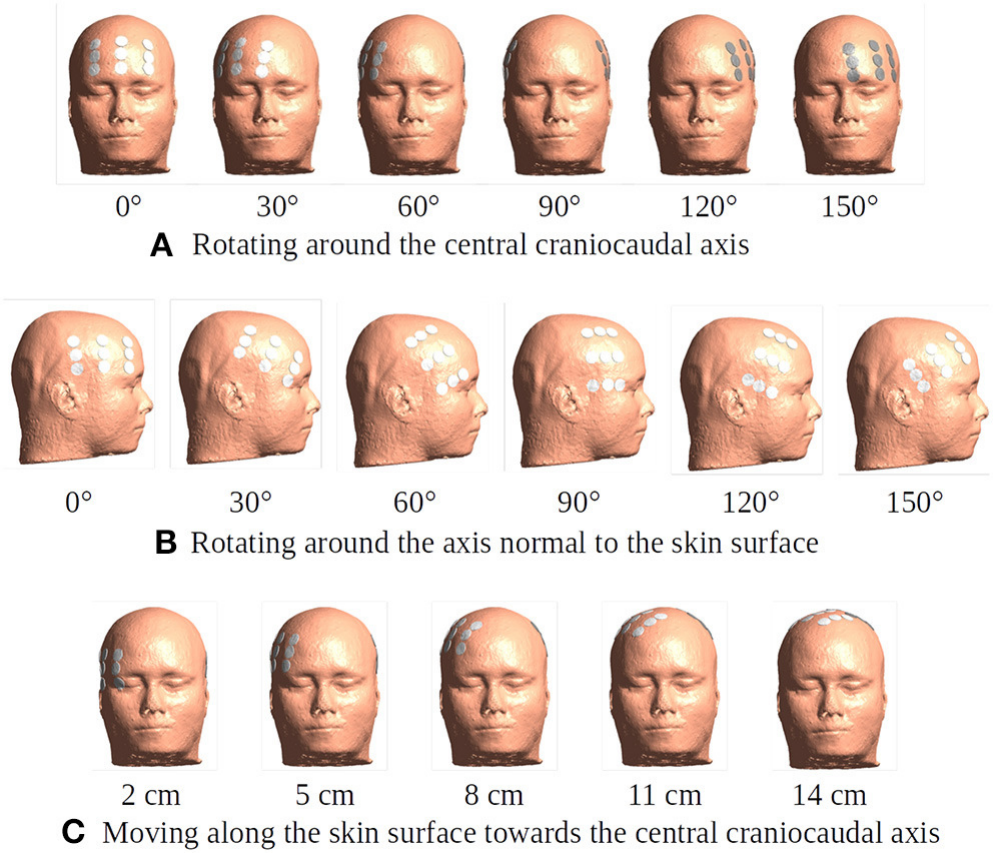
Different placement of the transducer arrays. The electrode arrays are shown in white and gray color placed in the opposite positions over the head model. **(A)** Rotation of the array pair in 15-degree intervals around the central craniocaudal *z*-axis. The rotation plane is 5 cm superior to the central horizontal plane. The subfigure illustrates six exemplary positions of degrees 0, 30, 60, 90, 120, and 150. **(B)** A 15-degree stepwise rotation of the ipsilateral array around the axis normal to the skin surface at the center of the array located above the resection cavity and residual tumor. The subfigure illustrates six exemplary positions of degrees 0, 30, 60, 90, 120, and 150. The contralateral array is maintained in the opposing position without rotation. The initial position is the same as the 60-degree one in **(A)**. **(C)** A 1-cm stepwise moving of the array pair along the skin surface toward the central craniocaudal axis. The starting position of the array pair is 3 cm inferior to the 60-degree one in **(A)**. This corresponds to 2, 3, 4, 5, 6, 7, 8, 9, 10, 11, 12, 13, 14, and 15 cm on the skin surface toward the central craniocaudal *z*-axis. The subfigure illustrates five exemplary positions.

To investigate the impact of array layout on TTFields intensity in the tumor, we evaluated a number of different clinically relevant positions for all burr hole configurations. Specifically, we calculated the field distribution for a single opposite array pair in the following situations:

1) Rotation of the array pair in 15 degree intervals around the central cranio-caudal *z*-axis (Figure 4A). We hypothesized that highest field intensities would be observed when the ipsilateral arrays overlapped the burr holes and lowest in the anterior-posterior position with no overlap and field lines oriented in parallel to the holes.
2) Rotation of the ipsilateral array around the normal to the skin surface at the center of the array located above the resection cavity (Figure 4B). The contralateral array was maintained in the opposing position without rotation. We hypothesized that the all rotated layouts would produce a reasonable field enhancement in the tumor, as the array was located close to the holes, although we expected stronger enhancement when the array overlapped the holes.
3) Translation of the array pairs along the central *Z*-axis toward the vertex of the head (Figure 4C). We expected upward translation to result in reduced global field intensity in the brain, as arrays were located increasingly close to each other resulting in a greater degree of shunting between them and less current penetrating into the brain. At the same time, we expected lower degrees of enhancement from burr holes as reduced overlap was introduced when electrodes were moved toward the vertex.

### Electric Field Calculation and Evaluations

Electric field calculations were performed using a development version of SimNIBS v4 at the Danish Research Center for Magnetic Resonance (DRCMR) and Technical University of Denmark (DTU) (Thielscher et al., 2015; Puonti et al., 2020). SimNIBS is an open-source software package and allows a realistic calculation of the electric field in an individual head model based on finite element method (FEM). Based on the pipeline of SimNIBS, we created the virtual SR-surgery on the skull of the head model using python 3.9 and also implemented an automated electric field simulation for different placements of the electrode arrays. Parameters of the TTFields modeling were set according to the clinical trial protocol (Mikic et al., 2021): the baseline-to peak current strength was set to 0.9 A and two 3 × 3 transducer arrays with electrodes of a height of 1 mm and diameter of 2 cm were used. The center-to-center distances between the electrodes was 45 × 22 mm.

According to our hypotheses, we used the electric field intensity to evaluate the TTFields dosimetry. The statistics of the electric field intensities were obtained as the median and peak values within four brain regions: WM, Gm, resection cavity and residual tumor. The calculation used the statistical library of NumPy v1.22 and the peak values were defined as the 99% percentile of the field intensities. The enhancement was defined as the difference between the field intensities with and without burr holes. The percentage of the enhancement was obtained by dividing the enhancement by the field intensities without burr holes.

### Uncertainty Quantification Analysis

We use the uncertainty quantification (UQ) approach provided in the SimNIBS toolbox to analyze the uncertainty of the electric field intensities in the brain due to uncertain knowledge of the ohmic conductivity of the tissue in the burrhole. The employed UQ approach is a regression-based generalized polynomial chaos method (gPC) (Saturnino et al., 2019) that requires the description of the uncertainty of the conductivity by a probability distribution. Here, we assumed that the conductivity of the burr holes lies somewhere between the conductivities of skin and CSF, and model its conductivity therefore as a uniform probability density over the interval [0.465 S/m, 1.654 S/m]. We assumed constant conductivities for the other tissues, see the corresponding values in Section Head Model Generation. We then used the UQ approach to estimate the probability density distribution of the electric field in the brain, given the uncertain conductivity of the burr holes. For each position in the brain, the mean and standard deviation of the estimated probability density distribution was determined for reporting.

## Results

### Effect of SR Surgery

**Figure 5:**
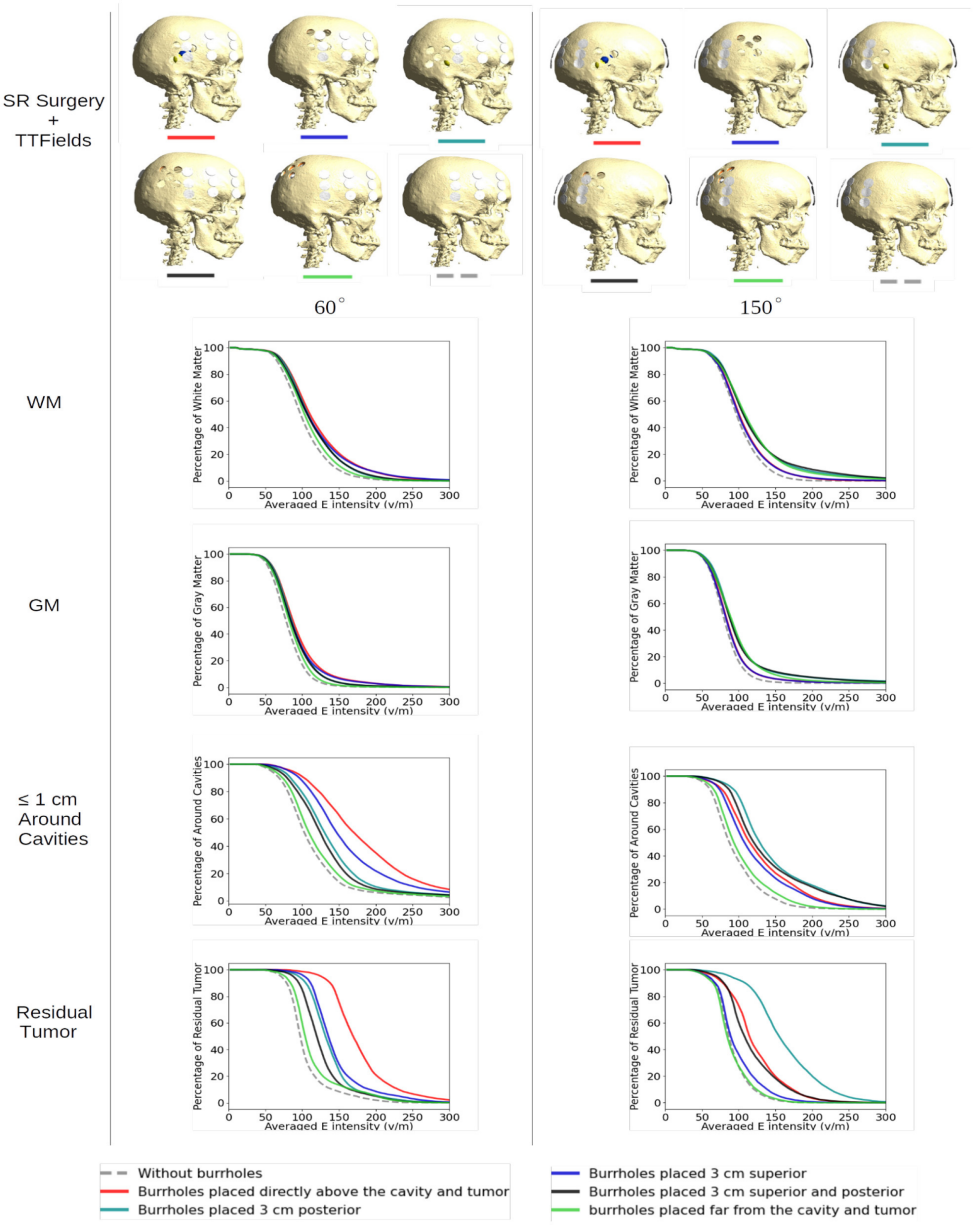
Effect of SR surgery with array positions at 60-degree (optimal) and 150-degree (suboptimal) rotations, respectively, for all configurations of SR surgery and the control without burr holes. The plots show the cumulative distributions of electrical field intensities obtained with burr holes over the resection cavity (red lines), the four alternative placements (other solid lines), and without burrholes (dashed gray lines). The cumulative distributions in the *y*-axis are given as the percentages of WM, GM, peri tumor, and residual tumor exposed to field intensities above the corresponding values on the *x*-axis.

Figure 5 shows array positions at 60-degree (optimal) and 150-degree (suboptimal) rotations, respectively, for all configurations of SR-surgery and “control” without cranial burr holes.

For both layouts, the control model exhibited the lowest field intensities in both WM, GM, tumor, and the peritumoral region (Figure 5, stippled line). This was expected given the shielding effect of the resistive skull. The range of median field intensities was 70–100 V/m.

For all SR-configurations, the dose-distributions remained relatively stable in the GM and WM tissues with <20 V/m median field enhancement. When the holes were placed far away from the tumor, neither layout was able to produce a benefit from SR-surgery in terms of field enhancement in the tumor or peritumoral regions, as expected. The most significant enhancement in the tumor (~70%) was observed when the burr-holes were located directly above the tumor with overlapping edge transducers of the array (60 degrees, Figure 5, bottom left panel, red). When there was no overlap, the field enhancement was lower (range 5–40%). Similar results were observed in the peritumoral region.

At the 150 degree rotation, the most significant enhancement occurred with 3 cm posterior displacement of the burr holes (95%). This was expected, given the more pronounced overlap between holes and transducers. Furthermore, the residual tumor was located in the posterior aspect of the resection cavity and therefore in close proximity to the burr holes. In the peritumoral region, all SR-configurations with holes close to the tumor bed were comparable in efficacy.

### Rotating Arrays Around the Head Circumference

**Figure 6:**
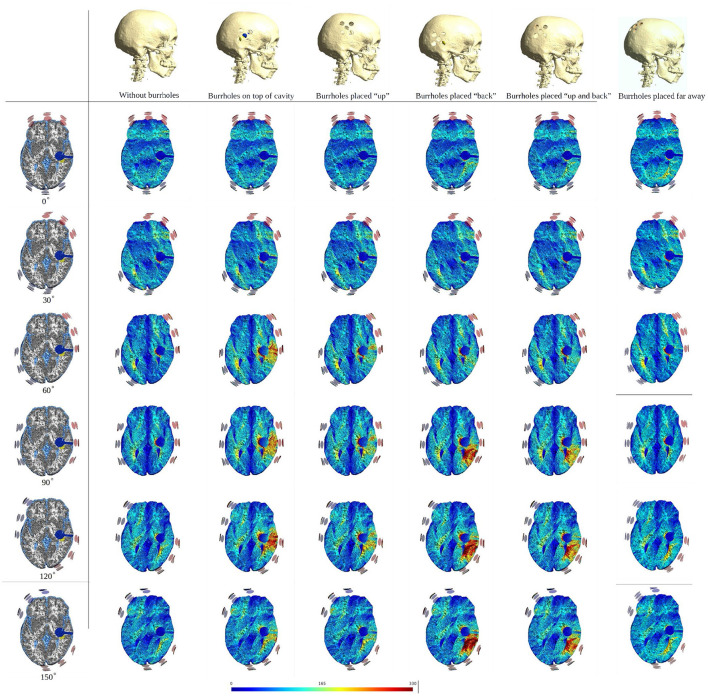
Axial view of TTFields intensity under array layouts 0°, 30°, 60°, 90°, 120°, and 150°. Different configurations of SR surgery and the control without burr holes are studied as illustrated in the top row. The first column presents an equivalent anatomical representation of the head model. The following two columns show a significant increase in field intensity when the burr holes are placed directly over the resection cavity and residual tumor, and the electrodes are placed close to or directly above the burr holes. Significant enhancement is also achieved with posterior displacement of the burr holes and slight rotation of arrays. Minor, limited field enhancement is observed when burr holes are placed far from the resection cavity and residual tumor. This experiment demonstrates how various burr hole placements impact the electric field in the resection cavity and residual tumor. Therefore, a recommended SR surgery configuration is to place burr holes directly above the resection cavity and residual tumor to maximize the electric field underneath.

To illustrate the effect of varying the array position relative to the burr hole configuration we included axial views of the topographical field distribution in the plane of the tumor and resection cavity for different combinations (Figure 6). The figure illustrates the concept that the field enhancement generally occurs in the tissue directly underlying the burr-holes and mainly when the array directly overlaps the holes. Furthermore, it is evident that superior displacement of the burr holes reduced the enhancing effect in the visualized plane.

**Figure 7:**
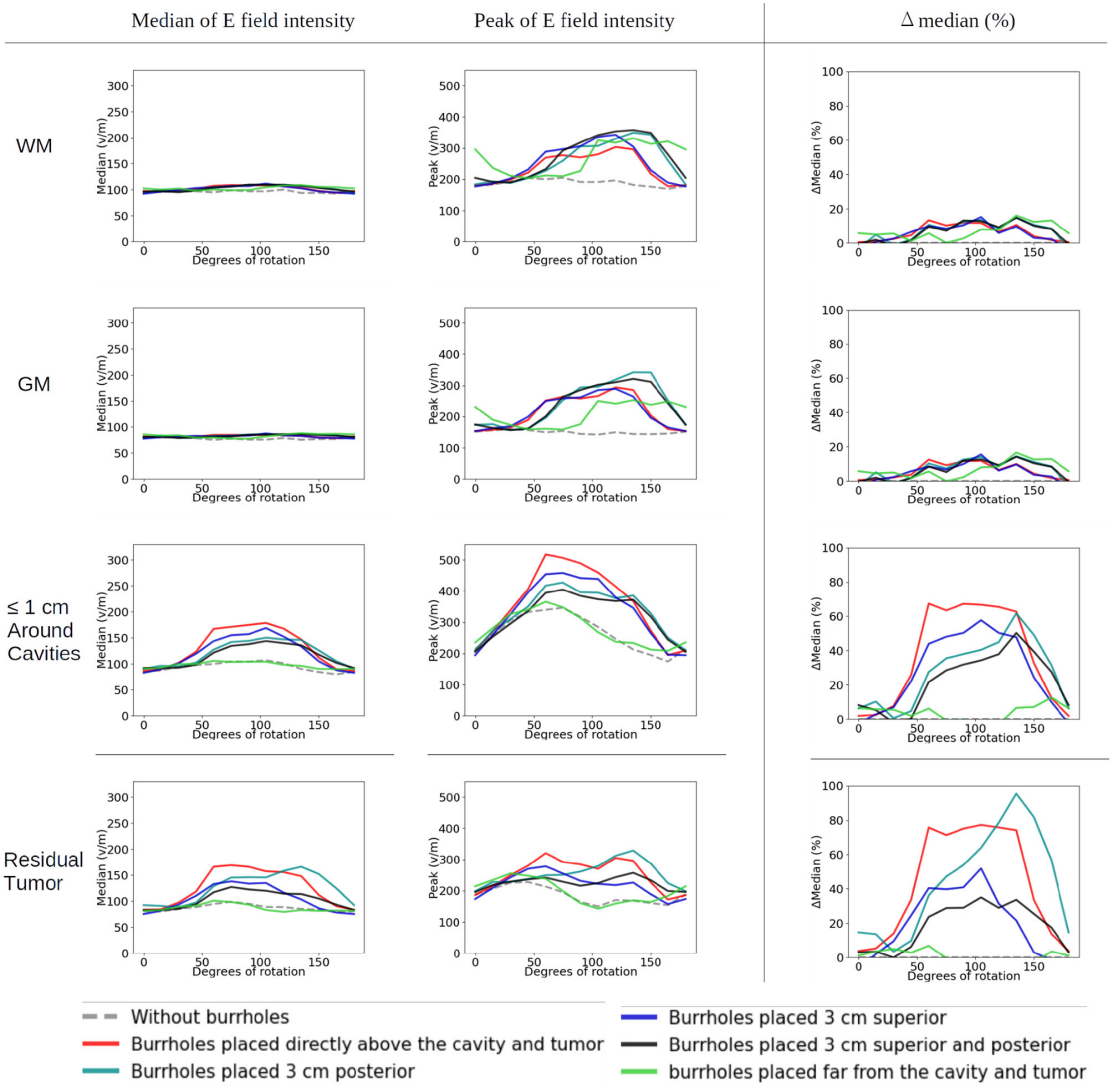
Statistical analysis of TTFields intensities with burr holes over the resection cavity and residual tumor (solid red lines), the four alternative positions (other solid lines), and the control with no burr holes (stippled gray line). The *x*-axis shows the rotation degrees around the central craniocaudal *z*-axis (Figures 4A, 8). The *y*-axis shows the field strength. The first two columns show median and peak field intensities for WM, GM, peri tumor and residual tumor, with all combinations of burr-hole configurations and rotational array layouts. The last column shows the enhancement of the field intensities when the SR-surgery is performed compared with the default configuration without burr holes.

The corresponding absolute median and peak field intensities are shown in Figure 7 for WM, GM, tumor, and peritumoral tissue and all combinations of burr-hole configurations and rotational array layouts. The figure also illustrates the field enhancements for these configurations relative to the default configuration without burr-holes.

Rotation around the head resulted in modest (~20 V/m) variation in the median field intensities in the tumor and peritumoral region, when no burr holes were present (absolute field range 90–120 V/m). However, the array layout had a significant impact on the observed peak fields in the peritumoral region, which ranged from 200 to 330 V/m. For all array positions, the median field intensities were relatively uniform around 80–100 V/m in WM and GM. Maximum median field intensities in the tumor occurred for layouts between 60 and 105 degrees when transducers were located close to the tumor.

As a general trend, all configurations of burr holes in the vicinity of the tumor region (i.e., except the distant location, Figure 3E) induced significant enhancement (30–100%) in the tumor and peritumoral region (Figure 7). Maximum efficacy was achieved for the central burr-hole configuration as described above although posterior displacement was also able to target the tumor efficiently with slightly rotated arrays. In general, array positions between 45 and 150 degrees caused considerable enhancement, while anterior-posterior configurations did not. This was expected due the lack of overlap between the array transducers and the holes in addition to the parallel direction of the induced field lines for the AP configurations relative to the holes in the skull. In the GM and WM tissues, we generally observed little enhancement of the field (<20%) for all array layouts and SR-configurations, which is expected as the fields were generally enhanced underneath the holes and not globally in the brain given dispersion of current with increasing distance from the holes.

**Figure 8:**
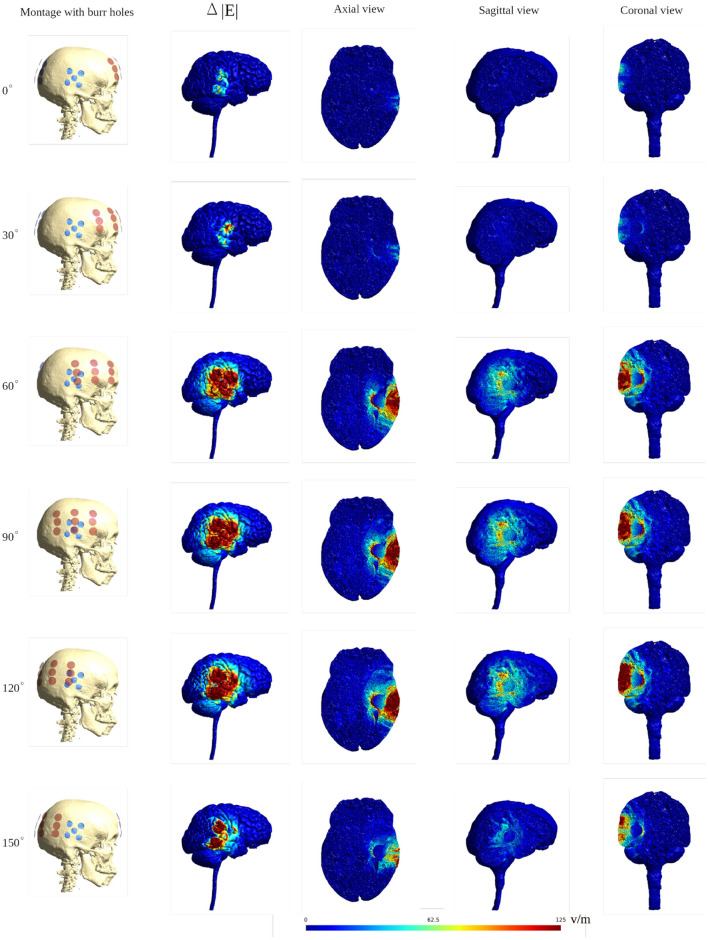
Heat map of the electric field intensity enhancement for the exemplary rotational layouts with burr holes placed directly over the resection cavity and residual tumor. Enhancement occurs right underneath the burr holes and mainly when active transducers are located close to or directly overlapped with the burr holes. The red dots and blue dots in the first column represent the placements of the transducers and the burr holes, respectively. The second column shows the electric field enhancement on the gray matter surface. The following three columns give all three topographical views of the field enhancement.

To further illustrate the concept of focal enhancement, Figure 8 shows topographical views of the absolute field enhancement (i.e., difference heat map) for all rotational layouts. Enhancement occurred directly underneath the holes, and mainly when active transducers were located in close proximity to or directly overlapped the burr holes. The enhancement occurs mainly in a 1–2 cm region around the exterior of the burr hole configuration and reaches ≈5–6 cm into the brain with particular enhancement in the deepest border of the resection cavity, which is perpendicular to the field lines. The Supplementary Videos 1, 2 show the animations of the changing of the field distributions for the array pair rotating around the central cranio-caudal *z*-axis. Supplementary Video 1 gives the enhancement of the field intensities on the gray matter surface, and Supplementary Video 2 shows the enhancement in the axial, sagittal, and coronal planes.

### Rotating Arrays Around Their Central Normal Axis

**Figure 9:**
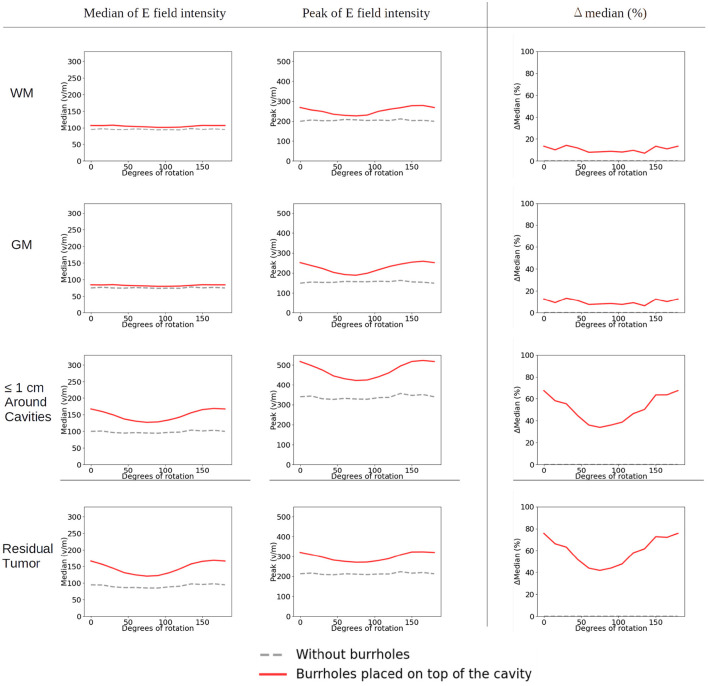
Statistical analysis of TTFields intensities with burr holes directly over the resection cavity and residual tumor (solid red lines) and without burr holes (dashed gray lines). The *x*-axis shows the rotation degrees of the ipsilateral array around the axis normal to the skin surface at the center of the array (Figures 4B, 10). The *y*-axis shows the field strength. The first two columns represent the median and peak values of TTFields intensities for the four tissue types (WM, GM, peri tumor and residual tumor). The last column shows the field intensity enhancement when SR-surgery is performed. The TTFields intensities of WM and GM are similar with or without burr holes. The field intensity in the peri tumor and the residual tumor decreases with electrodes with no/little overlap with the burr holes, corresponding to 60–105 degrees.

In the control situation with no burr holes, rotation of the ipsilateral arrays did not affect the field distribution significantly. The topographical distributions were close to identical and the median and peak intensity values were unaffected in all tissues (Figure 9, stippled line).

**Figure 10:**
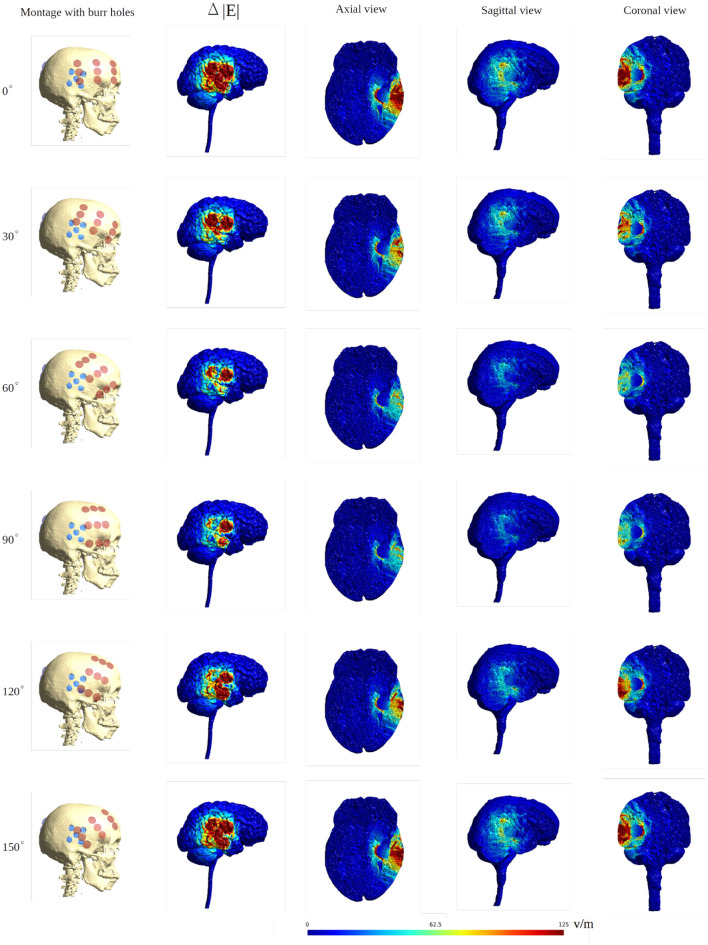
Heat map of the electric field intensity enhancement of the SR surgery, with the rotation of the ipsilateral array around the axis normal to the skin surface at the center of the array. The contralateral array is kept in the opposing position without rotation. The red and blue dots in the first column represent the placements of the electrodes and the burr holes. The second column gives the enhancement on the gray matter surface, and the last three columns show different views of the field enhancement.

When introducing burr holes in the optimal position, we observed a greater degree of variation in the field distribution between the rotated layouts in the tumor and peritumoral regions. Specifically, stronger field enhancement occurred underneath the burr holes with direct transducer overlap (Figure 10). The most effective layouts were those with greater degrees of overlap, i.e., 0, 15, 135, and 150 degrees. In the GM and WM tissues, rotation did not affect the field distribution notably, and the effects were mainly locoregional under the burr holes.

The Supplementary Videos 3, 4 show the animations of the changing of the field distributions for the array pair rotating around the normal to the skin surface at the center of the ipsilateral array overlapped with the burr holes. Supplementary Video 3 gives the field enhancement on the gray matter surface, and Supplementary Video 4 shows the enhancement in three different planes.

### Moving the Arrays Upwards

**Figure 11:**
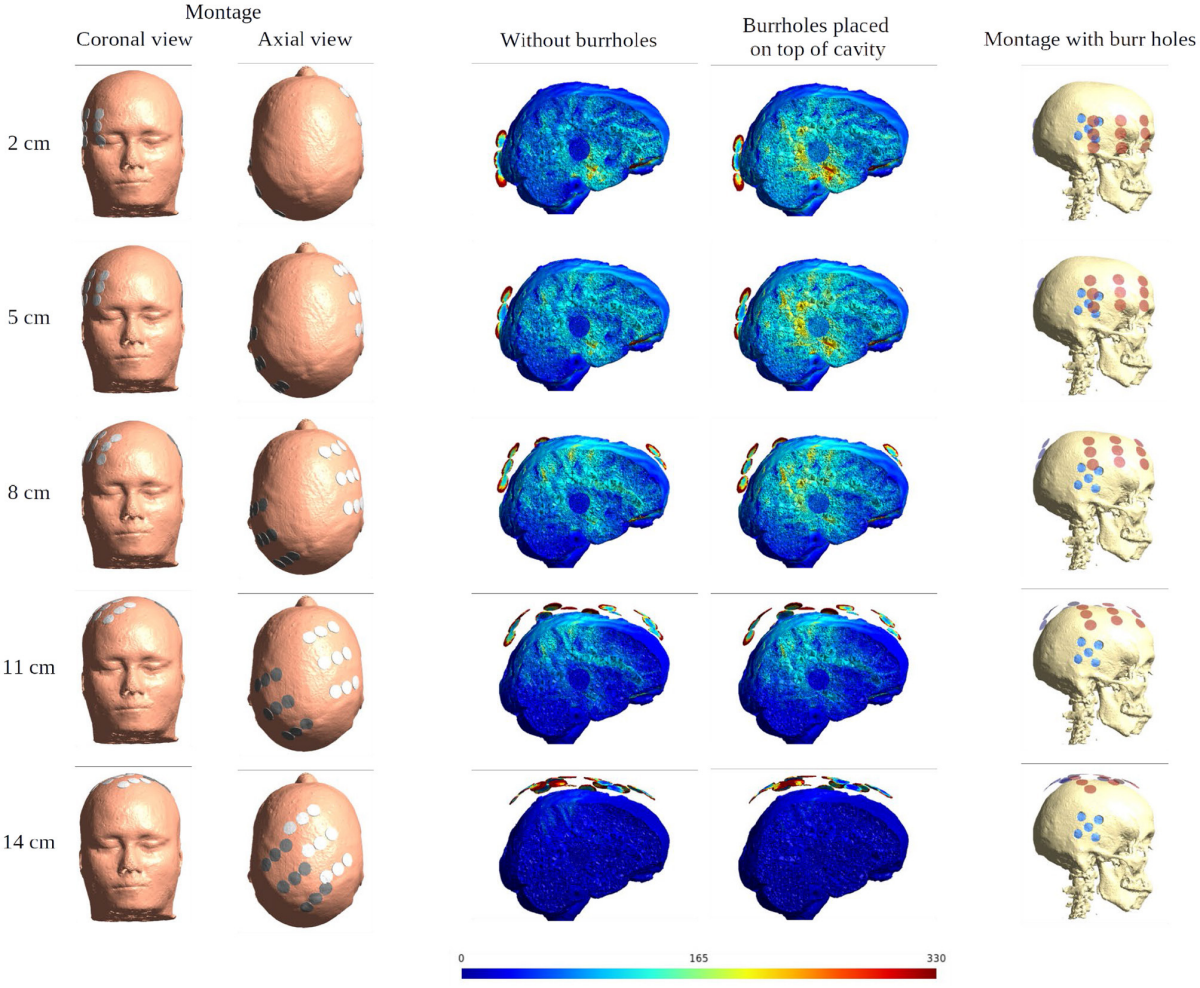
Effect of SR surgery with the moving of the array pair toward the central craniocaudal *z*-axis. The burr holes for the SR surgery are placed directly over the resection cavity and residual tumor. The first two columns show the electrode montage in different views. The following two columns give the sagittal view of the electric field intensities with and without burr holes. The last column presents the overlay of the electrode array (red) and the burr holes (blue) for the SR surgery configuration.

**Figure 12:**
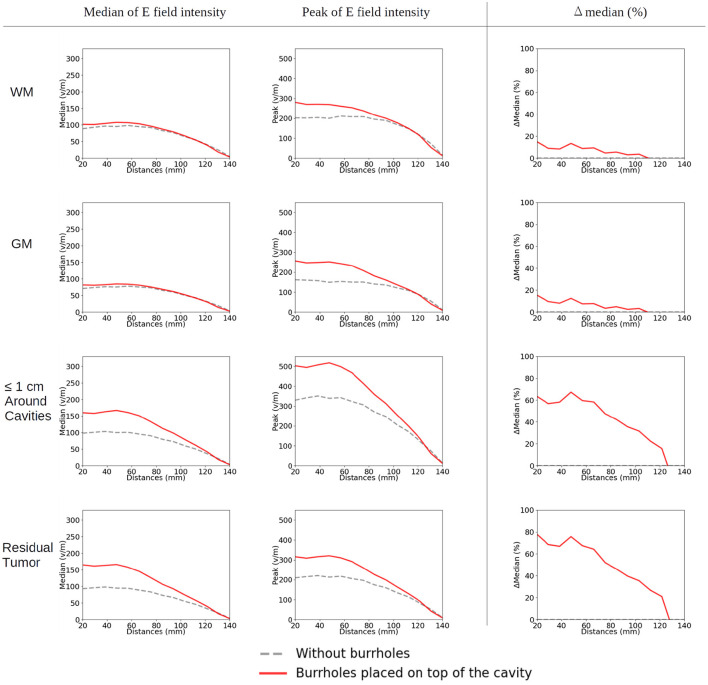
Statistical analysis of TTFields intensities with burr holes directly over the resection cavity and residual tumor (solid red lines) and without burr holes (dashed gray lines). The *x*-axis shows the moving of the arrays on the skin surface toward the central craniocaudal axis in millimeters (Figures 4C, 11, 13). The *y*-axis shows the field strength. The first two columns represent the median and peak values of TTFields intensities for the four tissue types (WM, GM, peri tumor, and residual tumor). The last column shows the field intensity enhancement when SR-surgery is performed. The observed median values of E field on both WM and GM show to be unaffected by burr holes. Namely, the red line with burr holes, and the gray line without burr holes, are close to each other or even overlap. Stronger shunting effects are observed when arrays are placed closer to each other (corresponding to 80–140 mm). The electric field drops to near-zero when the two arrays overlap (140 mm).

**Figure 13:**
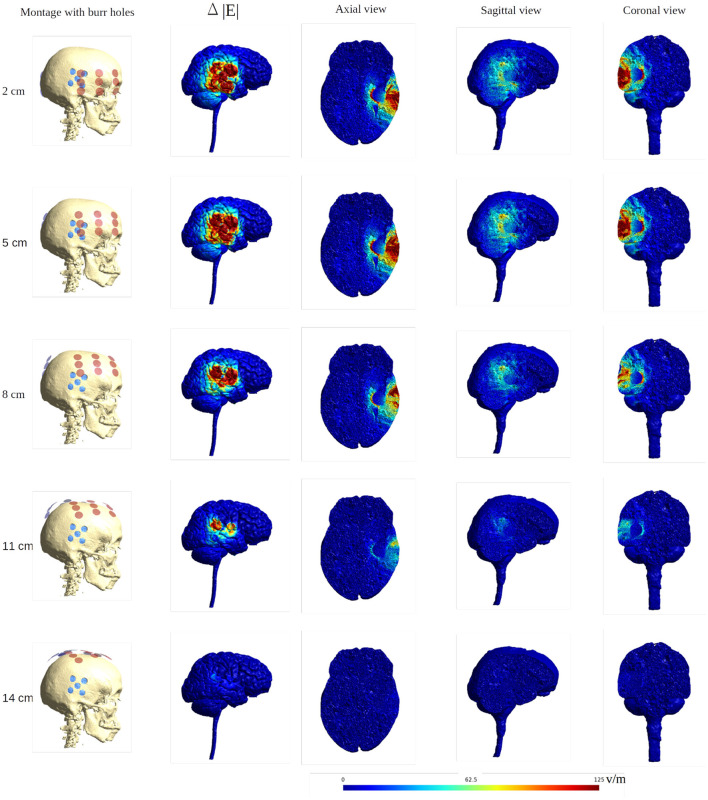
Heat map of the electric field intensity enhancement of the SR surgery, with the moving of the arrays toward the central craniocaudal axis. The positions of the electrode arrays were also shown in Figures 3C, 11 in different views. The red and blue dots in the first column represent the placements of the electrodes and the burr holes. The second column gives the enhancement on the gray matter surface, and the last three columns show all views of the E field enhancement.

When moving the arrays toward the vertex of the head, the field intensity progressively dropped in all tissues of the head, due to increased shunting of current through the skin between the electrodes (Figure 11). At the most extreme position at the top of the head, the field intensity was close to zero, illustrating the importance of maintaining the electrodes far apart on either side of the head. Equivalently, the relative enhancement caused by burr holes was also reduced with the *z*-axis translation from 60 to 80% in the optimal position (tumor and peri tumor) to 0% at the maximum translation, Figure 12. Again, enhancement occurred underneath the burr holes covered by active transducers, Figure 13. The Supplementary Videos 5, 6 show the animations of the changing of the field distributions for the array pair moving upwards to the vertex of the head. Supplementary Video 5 gives the enhancement on the gray matter surface, and Supplementary Video 6 shows the enhancement in axial, sagittal, and coronal planes.

### Uncertain Conductivity of the Burr Holes

**Figure 14:**
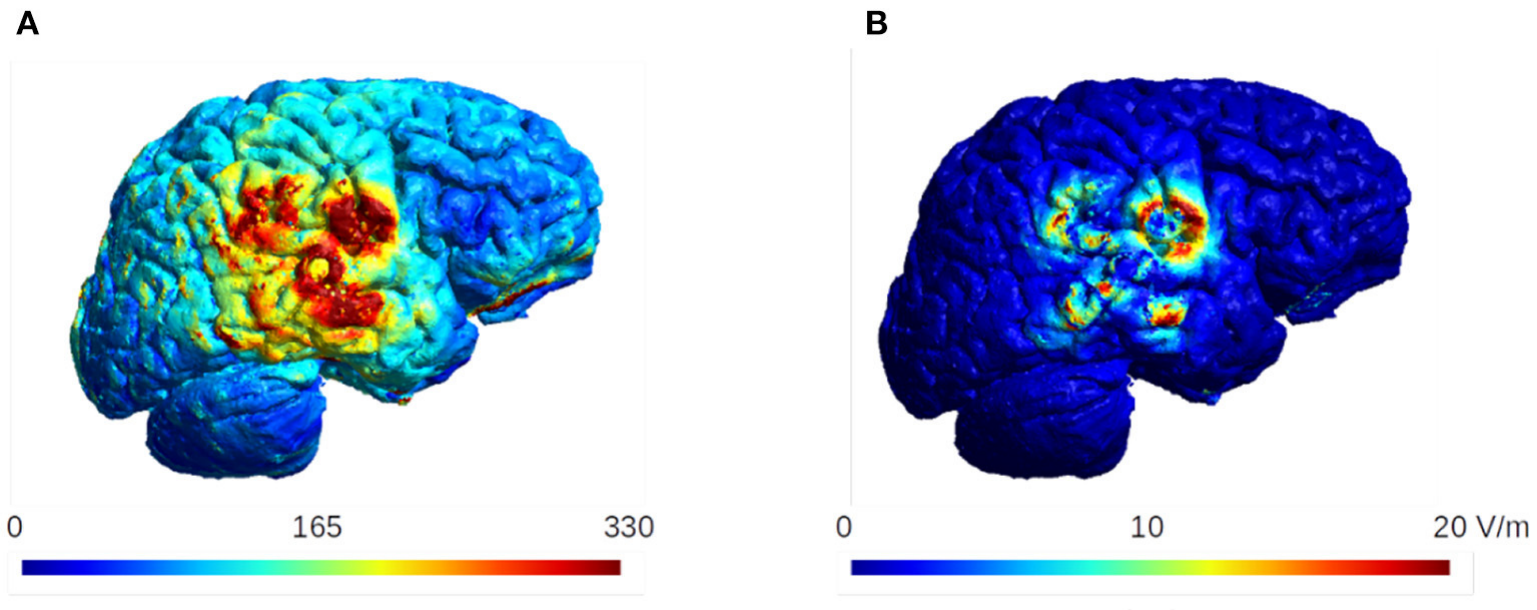
The mean and the standard deviation of the electric field intensity on the gray matter surface. The burr holes are located directly above the tumor and overlapped with the transducer array (60 degrees, Figure 8). These are the optimal configuration for the burr hole surgery and the optimal layout for the electrode arrays. The standard deviation is ~20 V/m in this case, which is <10% of the mean values in the region of interest.

Figure 14 shows the mean and the standard deviation of the electric field intensity on the gray matter surface when considering the uncertainty of the conductivity of the burr holes. The results are for the optimal burrhole position and electrode array layout. The burr holes are located directly above the tumor and overlapped with the transducer array (the row of 60 degrees, Figure 8).

The results indicate that the standard deviation does not exceed ~20 V/m, which is about 10% of the mean electric field strength in the region of interest. We therefore conclude that the effect of the uncertainty in the conductivity of the burr holes is small in our analysis of the TTFields dosimetry.

## Discussion

Recently, cranial burr holes have been shown to greatly increase the intensity of TTFields in underlying brain tissue. The concept is currently being tested in a randomized clinical phase 2 trial against recurrent glioblastoma. In this paper, we examined the influence of various configurations of burr holes in combination with a multitude of clinically relevant array layouts. Our aim was to provide clinicians with simple guidelines to be used when positioning the burr holes and transducer arrays in order to achieve maximum dose enhancement in the region of interest.

### Key Learnings

Our findings demonstrate the feasibility of a relatively simple set of guidelines to help surgeons plan and conduct SR-surgery and subsequent TTFields array planning. With these guidelines, significant field enhancement can be achieved in the underlying tumor, while absolute optimization would require extensive computation. It is evident that minor deviations and adjustments (<3 cm) of both burr holes configurations and layouts will still be able to induce a significant enhancement, leaving some margin of flexibility for the treating physician. Based on our findings, we suggest the following key principles to be followed when implementing SR-surgery with TTFields:

1) Field enhancement will occur mainly underneath the burr holes.
2) Burr holes should be placed in close proximity to the tumor or resection cavity, where a high field dose is desired. Slight displacement (up to 3 cm at least) will likely still produce enhancement although less.
3) Field enhancement will be most pronounced when array transducers directly overlap the burr holes. Ideally, ipsilateral transducers from both array pairs should overlap the holes. Correct array layout is highly important to maximize the benefit of the burr holes, while it is less important when no burr holes are present. Significant enhancement can be achieved with a wide range of array layouts as long as overlap or close proximity with the burr holes is maintained. Strongest fields occur underneath the array edges as previously (“edge effect”; Korshoej et al., 2018).
4) The contralateral array in a pair, should be placed far away from the ipsilateral array to avoid current shunting through the skin. Ideally, the two arrays in a pair should be placed on either side of the head rather than close to the top of the head to allow the current to pass through the head. Placement of arrays directly next to each other, completely diminishes the intracranial anti-tumor field.
5) Rotation of an array around its normal axis is less significant, when the position of the contralateral array is maintained. In the standard situation without holes this does not change the field distribution. When burr holes are present it is important that the principle of overlap between the holes and the arrays is respected.
6) The uncertainty quantification analysis quantifies the changes in the electric field intensity with respect to the conductivity variation in the burr holes. The results show that the effect of the uncertainty in the conductivity of the burr holes is minor in the simulation.

### Limitations

The results and conclusions in our study were based on a single computational head model created from a healthy person's MRI. Although this limits the generalizability of the conclusions in terms of absolute values, we believe the described principles are general in nature and apply widely to GBM models. However, it is important to bear in mind that factors such as tumor configuration, position, shape, size will greatly affect the regional conductivity and anatomy of the model and hence the resulting field distribution (Wenger et al., 2015a). Similar considerations apply for variations in head size and shape, peritumoral edema (Lang et al., 2020), and the thickness of the skull and subarachnoid space (Wenger et al., 2015a). For this reason, it would be preferable and ideal to employ a patient specific model based on a recent MRI to achieve numerically accurate results (Lok et al., 2017; Timmons et al., 2017). However, the patient specific model in our study has limitations. Korshoej et al. (2016) indicates that deep seated tumors are largely unaffected by SR-surgery and OptimalTTF-2 (Mikic et al., 2021) have specified inclusion criteria to ensure tumor locations that are best suited for burr holes. The patient data used for this study is from the OptimalTTF-2 trial and therefore the findings in this study may not translate to all patients. Furthermore, there is uncertainty in the literature of the conductive values in general but especially of the skull. The lower the conductive value assigned to the skull the more pronounced significance on E-field improvement from the burr holes. The value assigned (0.01 S/m) is within the range of what is “standard” (0.013 S/m) (Wenger et al., 2015a) and therefore we consider our results realistic. However, there is a possibility that the real conductive value is lower/higher which would impact the results of this study.

Also in clinical TTFields therapy, two pairs of arrays, each with a separate current source, are utilized and sequentially activated in a 50/50 duty cycle to induce orthogonal fields and thereby cover a larger topographical area and target cells dividing in random directions more efficiently (Korshoej and Thielscher, 2018b; Ballo et al., 2019; Korshoej, 2019; Korshoej et al., 2019). In our study, we systematically analyzed the field intensities of only a single active pair. Consequently, it should be noted that clinical treatment would be conducted with any feasible combination of layouts with the aim of maximizing the field intensity in the region of interest. I practice this would be achieved by combining layouts that are approximately orthogonal and that collectively produce the largest average field intensity (Korshoej et al., 2018).

Finally, it should be noted, which is a general limitation in TTFields modeling to date, is that we did not model the small (1–2 mm) craniotome track created during surgery nor the cranial fixation clamp device, which is made from titanium. Arguably, both aspects could greatly affect the E-field as they would likely contribute to current shunting through these corridors and thereby increase the underlying fields intensity. However, given the complexity of modeling these variable aspects, we believe this topic is beyond the scope of this paper and rather merits a separate in-depth investigation.

### Future Perspectives

Future studies based on personalized patient models would be required to validate the numerical accuracy of our findings. These studies should use post-operative MRIs from patients undergoing SR-surgery and subsequent TTFields, such as in the OptimalTTF-2 trial (Mikic et al., 2021). Furthermore, it would be highly valuable to do repeated assessments of the field at progression to evaluate the correlation between field dose and topographical recurrence patterns. In order to process patient scans efficiently, faster and automatized computational pipelines are needed.

## Data Availability Statement

The original contributions presented in the study are included in the article/Supplementary Material, further inquiries can be directed to the corresponding author/s.

## Ethics Statement

The studies involving human participants were reviewed and approved by Central Denmark Region Committee for Health Research Ethics (68928/1-10-72-214-19). The patients/participants provided their written informed consent to participate in this study.

## Author Contributions

Model creation and data analysis was conducted by FC supervised by AT. The manuscript draft was led by AK and FC, with contributions from NM, and the final manuscript was edited and approved by all. Figures were created mainly by FC with contributions from NM and adapted by AK. The study outline was planned by AK and AT. All authors contributed to the article and approved the submitted version.

## Funding

Funding has been received from the Danish Cancer Society by 1.700.00 DKK (Grant No. R322-A17630), Independent Research Fund Denmark by 1.392.683 DKK (Grant No. 9039-00307B), and Aarhus University, Department of Clinical Medicine by 550.00 DKK. This study has also received funding from the European Union's Horizon 2020 research and innovation programme under grant agreement No. 731827 (STIPED). Furthermore, this study was supported by the Lundbeck foundation (Grant No. R313-2019-622) and the NIH (Grant No. 1RF1MH117428-01A1). The results and conclusions in this article present the authors' own views and do not reflect those of the EU Commission.

## Conflict of Interest

The authors declare that the research was conducted in the absence of any commercial or financial relationships that could be construed as a potential conflict of interest.

## Publisher's Note

All claims expressed in this article are solely those of the authors and do not necessarily represent those of their affiliated organizations, or those of the publisher, the editors and the reviewers. Any product that may be evaluated in this article, or claim that may be made by its manufacturer, is not guaranteed or endorsed by the publisher.

## Supplementary Material

Supplementary Video 1 Animation of changes of field distributions for the array pair rotating around the central craniocaudal *z*-axis. From left to right, the images show the field intensities without burr holes, with burr holes, and enhancement on the gray matter surface.

Supplementary Video 2 Animations of changes of the field distributions for the array pair rotating around the central craniocaudal *z*-axis. The leftmost image presents the montage of the electrode over the skull. The electrode arrays are illustrated in red and the burr holes in blue. The other three images show the enhancement of the electric field in the axial, sagittal, and coronal planes.

Supplementary Video 3 Animation of changes of field distributions for the ipsilateral array rotating around the axis normal to the skin surface at the center of the array located above the resection cavity and residual tumor. The contralateral array is maintained in the opposing position without rotation. From left to right, the images show the field intensities without burr holes, with burr holes, and enhancement on the gray matter surface.

Supplementary Video 4 Animations of changes of the field distributions for the ipsilateral array rotating around the axis normal to the skin surface at the center of the array located above the resection cavity and residual tumor. The leftmost image presents the montage of the electrode over the skull. The electrode arrays are illustrated in red and the burr holes in blue. The other three images show the enhancement of the electric field in the axial, sagittal, and coronal planes.

Supplementary Video 5 Animation of changes of field distributions for the array pair moving on the skin surface toward the central craniocaudal *z*-axis. From left to right, the images show the field intensities without burr holes, with burr holes, and enhancement on the gray matter surface.

Supplementary Video 6 Animations of changes of the field distributions for the array pair moving on the skin surface toward the central craniocaudal *z*-axis. The leftmost image presents the montage of the electrode over the skull. The electrode arrays are illustrated in red and the burr holes in blue. The other three images show the enhancement of the electric field in the axial, sagittal, and coronal planes.

## References

[B1] “Ernie”. Available online at: https://simnibs.github.io/simnibs/build/html/dataset.html~(accessed).

[B2] Ballo M. T. Urman N. Lavy-Shahaf G. Grewal J. Bomzon Z. Toms S. (2019). Correlation of tumor treating fields dosimetry to survival outcomes in newly diagnosed glioblastoma: a large-scale numerical simulation-based analysis of data from the phase 3 EF-14 randomized trial. Int. J. Radiat. Oncol. Biol. Phys. 104, 1106–1113. 10.1016/j.ijrobp.2019.04.008 31026557

[B3] Kirson E. D. Gurvich Z. Schneiderman R. Dekel E. Itzhaki A. Wasserman Y.. (2004). Disruption of cancer cell replication by alternating electric fields. Cancer Res. 64, 3288–3295. 10.1158/0008-5472.CAN-04-0083 15126372

[B4] Korshoej A. R. (2019). Estimation of TTFields intensity and anisotropy with singular value decomposition: a new and comprehensive method for dosimetry of TTFields, in Brain and Human Body Modeling: Computational Human Modeling at EMBC 2018, eds Makarov S. Horner M. Noetscher G.. Cham: Springer. 173–193. 10.1007/978-3-030-21293-3_10 31725246

[B5] Korshoej A. R. Hansen F. L. Mikic N. Von Oettingen G. Sørensen J. C. H. Thielscher A. (2018). Importance of electrode position for the distribution of tumor treating fields (TTFields) in a human brain. Identification of effective layouts through systematic analysis of array positions for multiple tumor locations. PLoS ONE 13:e0201957. 10.1371/journal.pone.0201957 30133493 PMC6104980

[B6] Korshoej A. R. Hansen F. L. Thielscher A. Von Oettingen G. B. Sørensen J. C. H. (2017). Impact of tumor position, conductivity distribution and tissue homogeneity on the distribution of tumor treating fields in a human brain: a computer modeling study. PLoS ONE 12:e0179214. 10.1371/journal.pone.0179214 28604803 PMC5467909

[B7] Korshoej A. R. Lukacova S. Lassen-Ramshad Y. Rahbek C. Severinsen K. E. Guldberg T. L.. (2020). OptimalTTF-1: enhancing tumor treating fields therapy with skull remodeling surgery. A clinical phase I trial in adult recurrent glioblastoma. Neurooncol. Adv. 2, vdaa121. 10.1093/noajnl/vdaa121 33215088 PMC7660275

[B8] Korshoej A. R. Saturnino G. B. Rasmussen L. K. Von Oettingen G. Sørensen J. C. Thielscher A. (2016). Enhancing predicted efficacy of tumor treating fields therapy of glioblastoma using targeted surgical craniectomy: a computer modeling study. PLoS ONE 11:e0164051. 10.1371/journal.pone.0164051 27695068 PMC5047456

[B9] Korshoej A. R. Sørensen J. C. H. Von Oettingen G. Poulsen F. R. Thielscher A. (2019). Optimization of tumor treating fields using singular value decomposition and minimization of field anisotropy. Phys. Med. Biol. 64, 04nt03. 10.1088/1361-6560/aafe54 30641498

[B10] Korshoej A. R. Thielscher A. (2018a). Estimating the intensity and anisotropy of tumor treating fields using singular value decomposition. Towards a more comprehensive estimation of anti-tumor efficacy, in Annual International Conference of the IEEE Engineering in Medicine and Biology Society (EMBC), 2018, 4897–4900. 10.1109/EMBC.2018.8513440 30441441

[B11] Korshoej A. R. Thielscher A. (2018b). Estimating the intensity and anisotropy of tumor treating fields using singular value decomposition. Towards a more comprehensive estimation of anti-tumor efficacy, in 2018 40th Annual International Conference of the IEEE Engineering in Medicine and Biology Society (EMBC), 18–21 July 2018, 4897–4900. 30441441 10.1109/EMBC.2018.8513440

[B12] Lang S. T. Gan L. S. McLennan C. Monchi O. Kelly J. J. P. (2020). Impact of peritumoral edema during tumor treatment field therapy: a computational modelling study. IEEE Trans. Biomed. Eng. 67, 3327–3338. 10.1109/TBME.2020.2983653 32286953

[B13] Lok E. San P. Hua V. Phung M. Wong E. T. (2017). Analysis of physical characteristics of Tumor Treating Fields for human glioblastoma. Cancer Med. 6, 1286–1300. 10.1002/cam4.1095 28544575 PMC5463092

[B14] Lok E. San P. Liang O. White V. Wong E. T. (2020). Finite element analysis of Tumor Treating Fields in a patient with posterior fossa glioblastoma. J. Neurooncol. 147, 125–133. 10.1007/s11060-020-03406-x 31989489 PMC7076058

[B15] Lok E. San P. Wong E. T. (2019). Insights from computer modeling: analysis of physical characteristics of glioblastoma in patients treated with tumor-treating fields, in Brain and Human Body Modeling: Computational Human Modeling at EMBC 2018, eds Makarov S. Horner M. Noetscher G.. Cham: Springer. 155–161. 10.1007/978-3-030-21293-3_8 31725250

[B16] Mikic N. Korshoej A. R. (2021). Improving tumor-treating fields with skull remodeling surgery, surgery planning, and treatment evaluation with finite element methods, in Brain and Human Body Modeling 2020: Computational Human Models Presented at EMBC 2019 and the BRAIN Initiative^®^ 2019 Meeting, eds Makarov S. N. Noetscher G. M. Nummenmaa A.. Cham: Springer. 63–77. 10.1007/978-3-030-45623-8_4 32966031

[B17] Mikic N. Poulsen F. R. Kristoffersen K. B. Laursen R. J. Guldberg T. L. Skjøth-Rasmussen J.. (2021). Study protocol for OptimalTTF-2: enhancing Tumor Treating Fields with skull remodeling surgery for first recurrence glioblastoma: a phase 2, multi-center, randomized, prospective, interventional trial. BMC Cancer 21:1010. 10.1186/s12885-021-08709-4 34503460 PMC8427888

[B18] Miranda P. C. Mekonnen A. Salvador R. Basser P. J. (2014). Predicting the electric field distribution in the brain for the treatment of glioblastoma. Phys. Med. Biol. 59, 4137–4147. 10.1088/0031-9155/59/15/4137 25003941 PMC4137229

[B19] Nabors L. B. Portnow J. Ahluwalia M. Baehring J. Brem H. Brem S.. (2020). Central nervous system cancers, version 3.2020, NCCN Clinical Practice Guidelines in Oncology. J. Natl. Compr. Canc. Netw. 18, 1537–1570. 10.6004/jnccn.2020.0052 33152694

[B20] Novocure (2021). Trial pipeline [Online]. Available online at: https://www.novocure.com/our-pipeline/~(accessed).

[B21] Opitz A. Paulus W. Will S. Antunes A. Thielscher A. (2015). Determinants of the electric field during transcranial direct current stimulation. Neuroimage 109, 140–150. 10.1016/j.neuroimage.2015.01.033 25613437

[B22] Puonti O. Van Leemput K. Saturnino G. B. Siebner H. R. Madsen K. H. Thielscher A. (2020). Accurate and robust whole-head segmentation from magnetic resonance images for individualized head modeling. Neuroimage 219, 117044. 10.1016/j.neuroimage.2020.117044 32534963 PMC8048089

[B23] Saturnino G. B. Thielscher A. Madsen K. H. Knösche T. R. Weise K. (2019). A principled approach to conductivity uncertainty analysis in electric field calculations. NeuroImage 188, 821–834. 10.1016/j.neuroimage.2018.12.053 30594684

[B24] Taal W. Oosterkamp H. M. Walenkamp A. M. Dubbink H. J. Beerepoot L. V. Hanse M. C.. (2014). Single-agent bevacizumab or lomustine versus a combination of bevacizumab plus lomustine in patients with recurrent glioblastoma (BELOB trial): a randomised controlled phase 2 trial. Lancet Oncol. 15, 943–953. 10.1016/S1470-2045(14)70314-6 25035291

[B25] Thielscher A. Antunes A. Saturnino G. B. (2015). Field modeling for transcranial magnetic stimulation: a useful tool to understand the physiological effects of TMS?, in 2015 37th Annual International Conference of the IEEE Engineering in Medicine and Biology Society (EMBC), 25–29 August 2015, 222–225. 10.1109/EMBC.2015.7318340 26736240

[B26] Timmons J. J. Lok E. San P. Bui K. Wong E. T. (2017). End-to-end workflow for finite element analysis of tumor treating fields in glioblastomas. Phys. Med. Biol. 62, 8264–8282. 10.1088/1361-6560/aa87f3 29023236

[B27] Toms S. A. Kim C. Y. Nicholas G. Ram Z. (2019). Increased compliance with tumor treating fields therapy is prognostic for improved survival in the treatment of glioblastoma: a subgroup analysis of the EF-14 phase III trial. J. Neurooncol. 141, 467–473. 10.1007/s11060-018-03057-z 30506499 PMC6342854

[B28] Wagner T. A. Zahn M. Grodzinsky A. J. Pascual-Leone A. (2004). Three-dimensional head model simulation of transcranial magnetic stimulation. IEEE Trans. Biomed. Eng. 51, 1586–1598. 10.1109/TBME.2004.827925 15376507

[B29] Wenger C. Miranda P. C. Salvador R. Thielscher A. Bomzon Z. Giladi M.. (2018). A Review on Tumor-Treating Fields (TTFields): clinical implications inferred from computational modeling. IEEE Rev. Biomed. Eng. 11, 195–207. 10.1109/RBME.2017.2765282 29993870

[B30] Wenger C. Salvador R. Basser P. J. Miranda P. C. (2015a). The electric field distribution in the brain during TTFields therapy and its dependence on tissue dielectric properties and anatomy: a computational study. Phys. Med. Biol. 60, 7339–7357. 10.1088/0031-9155/60/18/7339 26350296 PMC4628548

[B31] Wenger C. Salvador R. Basser P. J. Miranda P. C. (2015b). Modeling Tumor Treating fields (TTFields) application within a realistic human head model, in Annual International Conference of the IEEE Engineering in Medicine and Biology Society (EMBC) 2015, 2555–2558. 10.1109/EMBC.2015.7318913 26736813

